# Rapid oxygen diffusion during high temperature alteration of zircon

**DOI:** 10.1038/s41598-018-22016-2

**Published:** 2018-02-26

**Authors:** Nick M. W. Roberts, Qiong-Yan Yang, M. Santosh

**Affiliations:** 1 NERC Isotope Geosciences Laboratory, British Geological Survey, Nottingham, NG12 5GG UK; 2 School of Earth Sciences and Resources, China University of Geosciences Beijing, 29 Xueyuan Road, Beijing, 100083 China; 3 Centre for Tectonics, Exploration and Research, University of Adelaide, Adelaide, SA 5005 Australia; 4 Department of Geology, Northwest University, Northern Taibai Str. 229, Xi’an, 710069 China

## Abstract

The mineral zircon through its isotopic and elemental signatures comprises the greatest archive recording the evolution of Earth’s continental crust. Recognising primary from secondary zircon compositional signatures is thus important for the accurate interpretation of this archive. We report two examples of metasedimentary rocks from high-grade shear zones within the Southern Granulite Belt of India, where anomalously high and homogeneous oxygen isotope signatures indicate disturbance of this isotopic system. Utilising the combined U-Pb-Hf-O and trace element signatures from these zircon grains, we postulate that fluid-assisted alteration has led to complete resetting of the oxygen isotope signatures. This case study presents a rarely observed natural example of potentially fast diffusion of oxygen under hydrous conditions. Given the pervasive nature of fluid interaction within high-grade and highly deformed rocks, we expect that such isotopic disturbance might be more common to nature than is currently reported. A lack of correlation between isotopic disturbance with cathodoluminescence or Th/U values, suggests that these altered zircon grains would not clearly be classified as metamorphic, in which case they would be expected to yield primary compositions. Caution is therefore advised when using detrital δ^18^O zircon compilations without a high level of scrutiny for primary versus secondary compositions.

## Introduction

Zircon (ZrSiO_4_) is the most abundantly used mineral for dating igneous and metamorphic events through geological history. In addition, the measurable fractionation recorded in the various isotopic systems it hosts provide a wealth of information on magmatic and metasomatic processes. After initial zircon formation, each isotopic system exhibits variable behavior during subsequent geological events. For example, post-crystallisation re-heating can induce lead-loss, a depletion of the daughter lead isotopes of the U-Pb decay chain. Due to their low diffusion rates^1–3^ and oxygen (^18^O/^16^O, referred to by δ^18^O) isotope systems are generally believed to be robust during subsequent magmatic reworking or metamorphic heating and burial, as long as the isotopes are preserved within non-metamict and non-altered zircon domains^4–10^. Dissolution-reprecipitation processes, typically occurring during high-grade metamorphism, will reset these isotopic systems within altered zircon domains^4,5,9^. Trace element signatures are also useful diagnostics within zircon, for example, Ti contents are used to determine zircon formation temperatures^11,12^. Most elements such as Ti and REE also have low diffusion rates^13,14^ and are believed to record primary signatures during zircon formation or reprecipitation, even during subsequent high-grade metamorphism. However, fluid-assisted alteration, both at low- and high-temperatures, and accelerated by metamictisation, forms a major caveat to the generally robust nature of most isotopic and elemental signatures that rely on slow diffusion rates. Because zircon from detrital records are now used widely to inform about Earth processes throughout geological history^15–20^, it is important to understand the processes and likelihood that the information recorded in these zircon grains reflects primary signatures. We report a case of disturbance of oxygen isotopes in zircon, using a combined *in-situ* zircon U-Pb, Lu-Hf, δ^18^O and trace element study of zircon grains in quartz-mica-schist from two adjacent high-grade shear zones within the Southern Granulite Terrane of south India.

## Geological Setting

**Figure 1:**
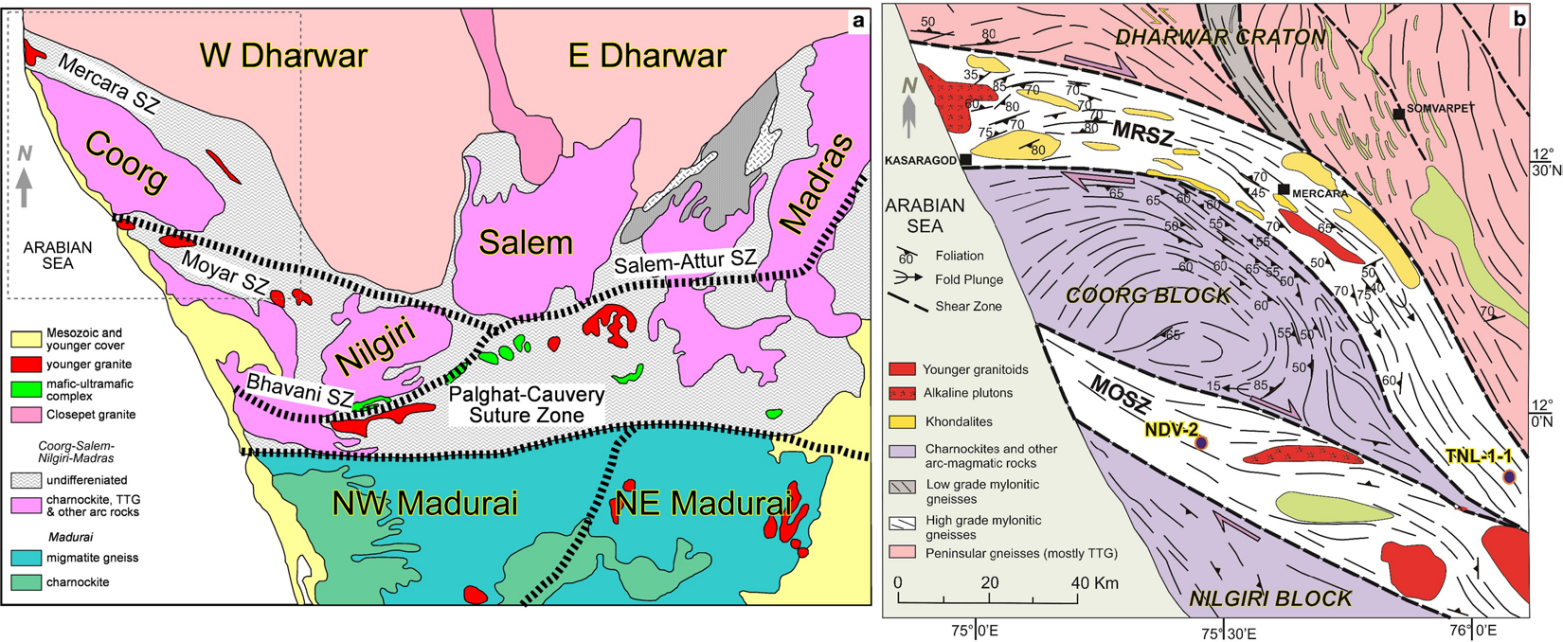
Geological sketch map of the northern part of the Southern Granulite Terrane showing the major crustal blocks and shear zones (modified after)^21,22^. (**b**) Geological sketch map of the Coorg Block and surrounding Mercara and Moyar Shear Zones (modified from Chetty *et al*.^59^, Jour. Geol. Soc. India, v.72, pp. 151–154; with permission from Geological Society of India, Bengaluru).

The Southern Granulite Terrane (SGT) forms the region of India south of the Dharwar Craton (Fig. 1), and is a mosaic of crustal blocks ranging in age from Mesoarchaean (3.2 Ga)^21,22^ up to late Neoproterozoic – Cambrian^23–25^. Among these, the Coorg Block is unique in preserving Mesoarchaean juvenile crust formation and lacking any imprints of the later Neoarchaean, Palaeoproterozoic and Neoproterozoic tectonothermal events that are widely represented in the surrounding crustal blocks in the SGT^21^. The margins of the Coorg Block and the bounding suture zones have been the focus of recent studies in preserving some of the oldest crustal remnants in Peninsular India^22^, and as the zone along which Mesoarchaean high grade metamorphism occurred, possibly associated with collisional orogeny^26^. The adjacent crustal blocks, Nilgiri, Biligiri Rangan, Salem and Madras (Fig. 1a), preserve common Neoarchaean crustal formation/recycling and metamorphism e.g.^27–30^. However, the Coorg Block is believed to be an exotic crustal domain within the SGT, in the absence of any records of the 2.5 Ga pervasive regional metamorphism^21,22^.

The Coorg Block is bound by the Moyar Shear Zone^21,31^ to the south, and the Mercara Shear Zone^32^ to the north (Fig. 1b). The Mercara Shear Zone formed from the accretion of the Coorg Block to the Western Dharwar Craton, which is inferred to have occurred at 3.0 Ga based on zircon metamorphic ages^26^. Steep gravity gradients have been imaged beneath the Mercara Suture Zone, and are considered to mark underplated high-density mafic lower crust that has welded the Coorg Block with the Dharwar Craton^33^. Peak metamorphic conditions recorded in Mercara Shear Zone granulites are estimated at 10–12 kbar at 700–900 °C^26^.

The Moyar Shear Zone marks the boundary between the Nilgiri and Coorg Blocks in the west, and the Nilgiri Block with the Dharwar Craton in its eastern extent. In the Wynad region at the confluence of the Mercara and Moyar Shear Zones, peak pressure temperature conditions have been estimated in metagabbros at 10.6–11.8 kbar at 900 °C, with the timing of this metamorphism estimated at 2.36–2.22 Ga, and the origin of this metamorphism being ascribed to suturing of the Coorg and Nilgiri Blocks^34^. Ultramafic rocks in this region record peak metamorphism of 6.5–8.7 kbar at 720–840 °C^35^. Further along strike to the southeast, peak conditions are recorded in garnet-bearing quartzofeldspathic gneiss at 9–11 kbar at 900 °C^36^. These authors present a model whereby the Moyar Shear Zone is part of a doubly vergent orogen marking the suturing in this region of the Nilgiri Block and the Dharwar Craton.

Metasedimentary belts comprising pelite-rich and quartz-rich lithologies are found within both the Mercara and Moyar Shear Zones. TNL-1–1 is taken from a set of nearly E-W trending laminated, pale red to white quartz mica schist bands, ranging in width from several tens up to 500 m. The protolith of these rocks likely correspond to ferruginous sandstone. Several thin bands of medium to fine- grained, finely laminated, pale green to yellowish white quartz mica schist intercalated with calcareous schist are also exposed around the Tirunelli temple area. Weathered quartz mica schist interbanded with fuchsite quartzite is exposed on the western side of Mambaram, and continues in the Kuppadi reserve forest NW of Vengur. A small hillock with fine laminated garnet-bearing brownish quartz mica schist occurs at Neduvaloor along the Irikur-Thaliparamba road. Sample NDV-2 is taken from here, where quartz mica schist occurs in association with fuchsite and micaceous quartzite, talc-tremolite schist, meta-BIF, fuchsite quartzite and amphibolite. Quartz mica schist in association with greenish fuchsite quartzite and medium grained white quartzites are also exposed in other locations in the area including Tirunelly, Manandawady and Sulthan Bathery. Metamorphic garnet, rutile and paragonite within quartz-mica-schists^22^ attest to them experiencing at least amphibolite-facies metamorphism. The peak temperature of one sample studied here (NDV-2) was previously estimated at 710–760 °C^22^, and sillimanite in some quartz-muscovite schist units suggests pressures up to 8 kbar^22^.

## Samples

**Figure 2:**
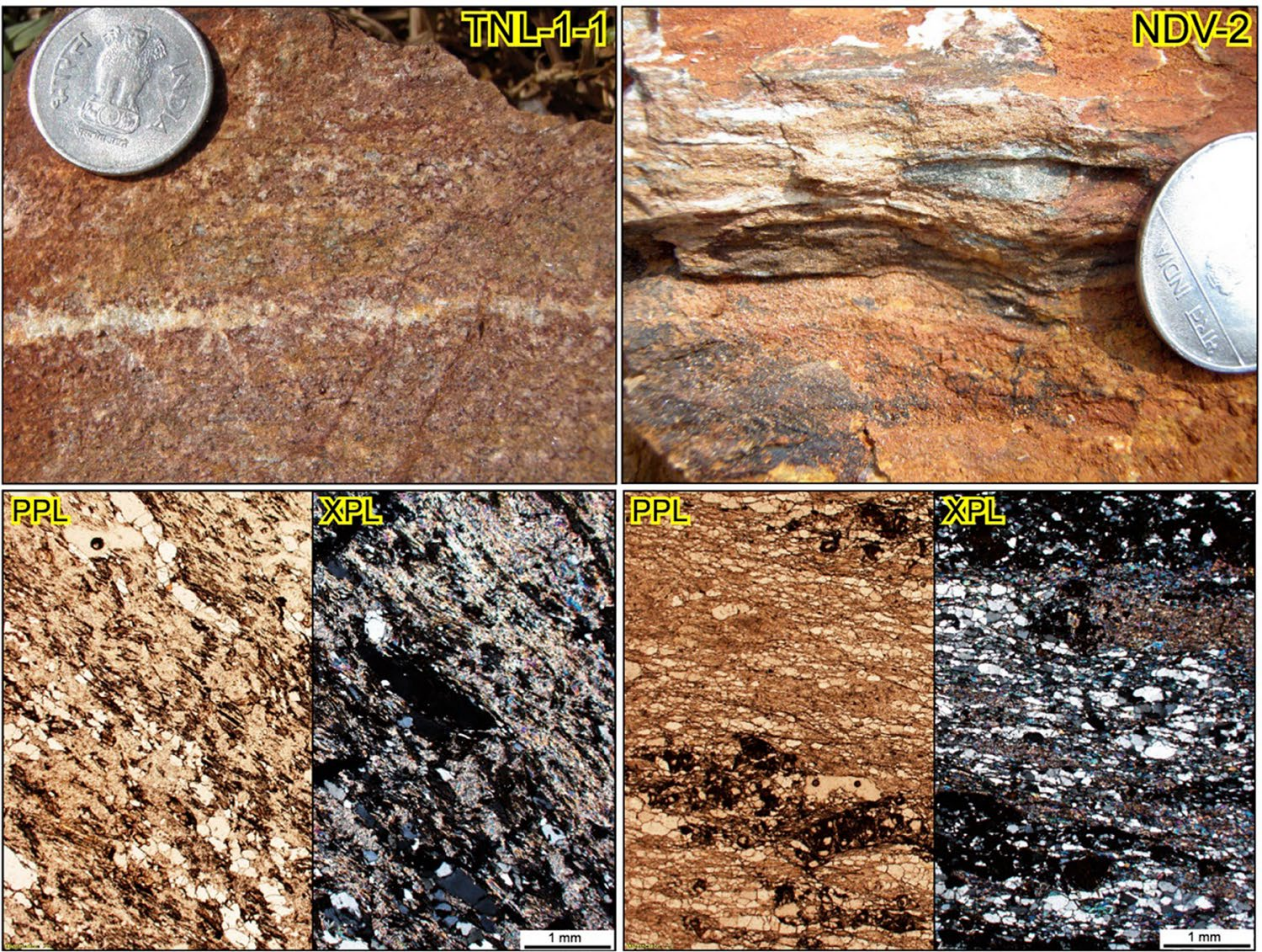
Field photographs and thin section microphotographs of samples TNL-1-1 and NDV-2. PPL = plane polarised light; XPL = cross polarised light.

**Figure 3:**
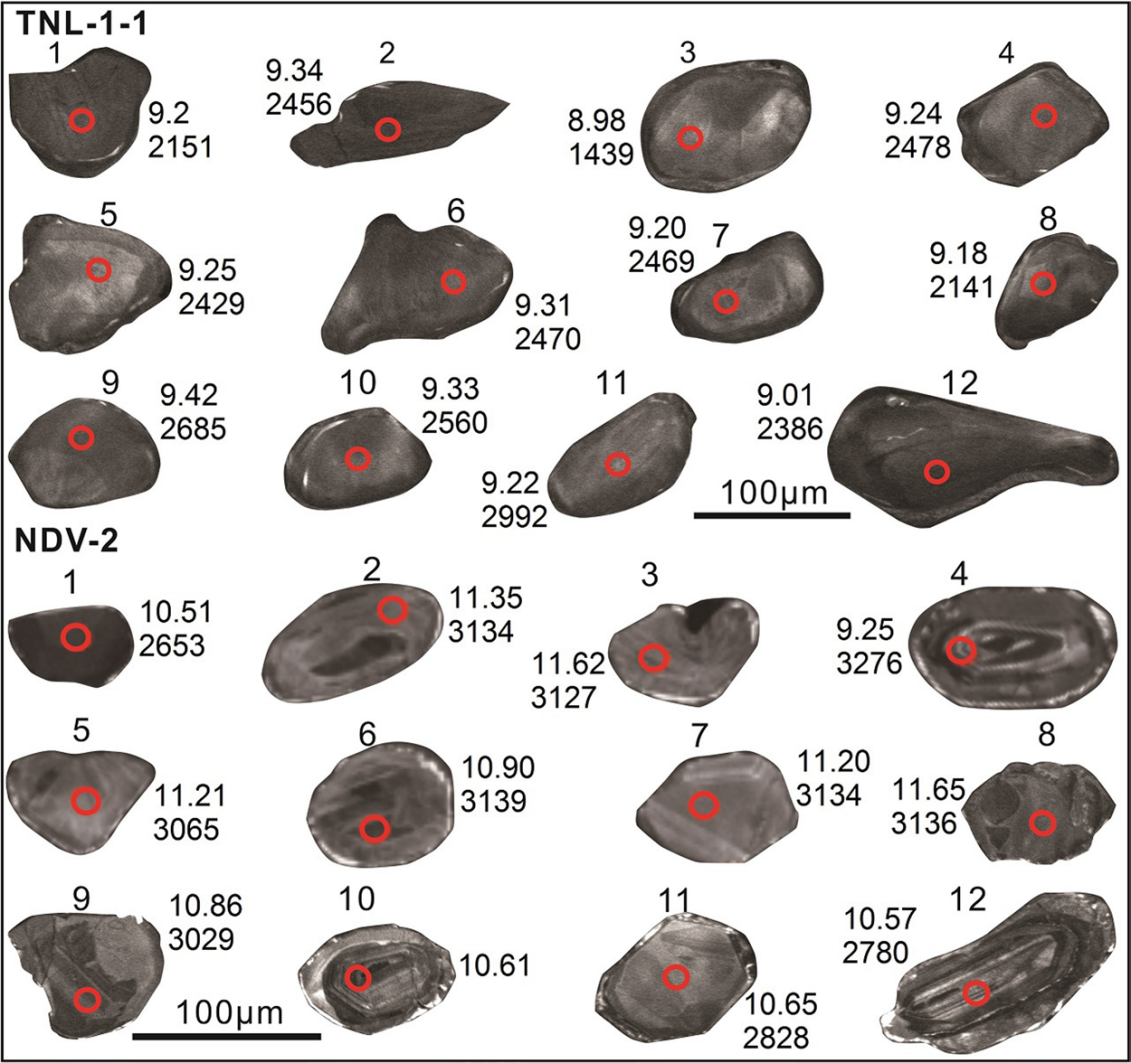
Cathodoluminescence (CL) image of zircon grains analysed for oxygen isotopes. The red spots show the location of the oxygen isotope analyses, and U-Pb and Hf isotope spots were placed directly over these. The δ^18^O (‰) and ^207^Pb/^206^Pb age (Ma) for each zircon is shown.

In this study, two representative samples of quartz mica schist (Figs 2 and 3) were analysed from both the Moyar and Mercara Shear zones. Sample TNL-1-1 (Mercara Shear Zone) from Tirunelli (GPS:N11°52′25.9″; E76°04′22.4″) shows prominent foliation defined by nematoblastic aggregates of white mica (<0.15 mm) and chlorite (<0.2 mm) together with elongated ribbons of quartz (0.1–1.9 mm). Sample TNL-1-1 is composed of muscovite/paragonite (80–90%) and quartz (10–20%) with accessory chlorite (altered biotite), rutile and zircon (Fig. 3). Zircon grains from TLN-1-1 (Fig. 3) are generally sub-rounded, and on average 100 to 200 μm long with 1:1 to 2:1 length:width ratios. The CL images show that most zircon grains have faint internal zoning with weak oscillatory or sector zonation patterns. Many zircon grains have thin rims that are too narrow for *in-situ* techniques employed here.

Sample NDV-2 from the Moyar Shear Zone (Fig. 2) is a garnet-bearing quartz mica schist from Neduvalloor (GPS: N12°03′16.7″; E75°27′33.9″) and shows a prominent foliation defined by the preferred alignment of muscovite and chlorite. The rock is dominantly composed of quartz (40–50%), muscovite (15–25%), chlorite (10–20%), garnet (5–10%), rutile (~1%) and zircon (Fig. 2). Except for garnet (~1.1 mm) and quartz (~2.2 mm), all the other minerals are fine grained (<0.3 mm). Zircon grains from NDV-2 (Fig. 3) are sub-rounded to anhedral, and on average 80 to 150 μm long with 1:1 to 2:1 length:width ratios. The CL images show that zircon grains have a range of internal textures, comprising strong to weak oscillatory, sector and faint patchy zonation.

## Results

### U-Pb

**Figure 4:**
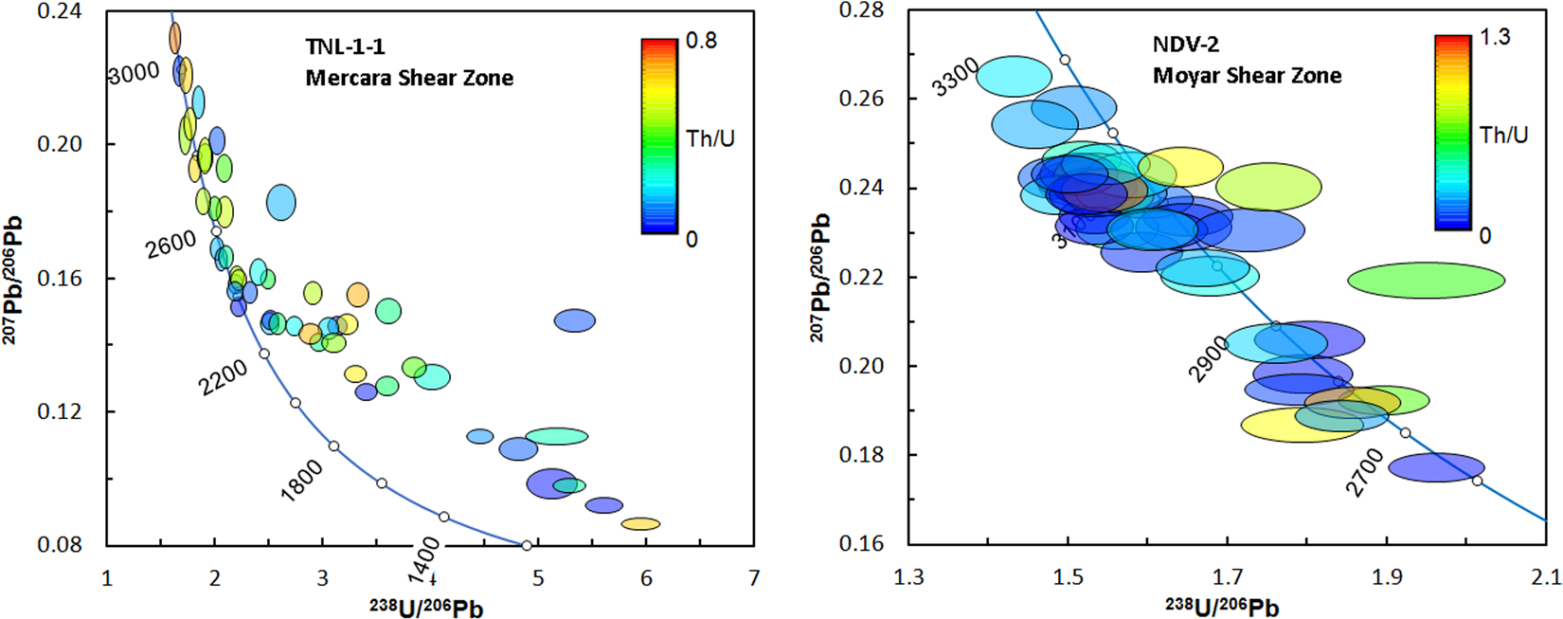
Tera-Wasserburg concordia diagrams for TNL-1-1 and NDV-2. Ellipses are 2σ and shaded according to the zircon Th/U values.

**Figure 5:**
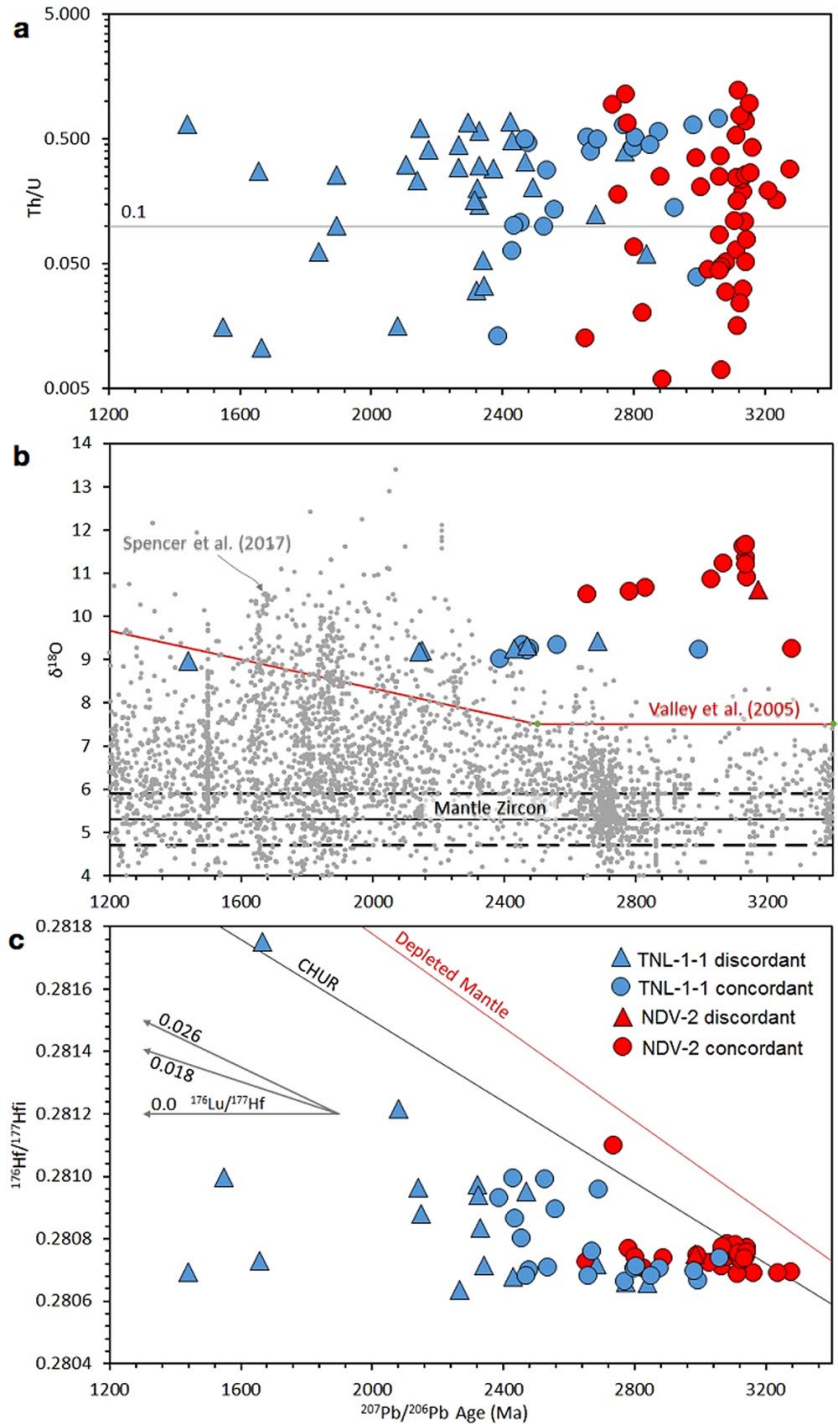
(**a**) Zircon Th/U compositions plotted according to the ^207^Pb/^206^Pb age (in Ma). Th/U of 0.1 is typically used for a discriminator of metamorphic zircon (<0.1) from magmatic zircon (>0.1)^60^. (**b**) Zircon δ^18^O compositions plotted according to ^207^Pb/^206^Pb age (Ma). Black bars represent mantle zircon composition of 5.3 ± 0.3‰^7^. The red line represents the global maximum value of zircon as described in^38^. The grey circles represent the most recent global compilation^52^. Note that the data presented here are substantially higher than zircon expected of this age. (**c**) Age corrected initial ^176^Hf/^177^Hf compositions against ^207^Pb/^206^Pb age (Ma). Depleted Mantle is that of^61^ and CHUR is that of^62^. Depicted are evolution lines for ^176^Lu/^177^Hf compositions of zero, i.e. lead-loss, and of average Large Igneous Province (LIP) crust and average island arc crust of 0.018 and 0.026 respectively, calculated from^63^.

TNL-1-1 exhibits an array of generally discordant data (Fig. 4), with ^207^Pb/^206^Pb ages of sub-concordant (>90% concordance) analyses ranging from 2386 to 3060 Ma. There is no precise lower intercept obtainable, since the data are scattered, reflecting variable discordance from a range of ~2.4 to 3.0 Ga primary ages. A chord drawn between the lowest concordant and most discordant ages regresses to a lower intercept of 802 ± 86 Ma with an upper intercept of 2497 ± 42 Ma. There is no particular correlation between age and Th/U contents; sub-concordant ages have a range of both ‘magmatic’ (>0.1) and ‘metamorphic’ (<0.1) values (Fig. 5a). NDV-2 exhibits an array of concordant to sub-concordant ages ranging from 2735 to 3212 Ma (Fig. 4), and clustering into two groups at ca. 2750–2900 Ga and ca. 3000–3150 Ga. There is no pattern linking Th/U content with age (Fig. 5a).

### δ^18^O

Oxygen isotope data are plotted against apparent age (^207^Pb/^206^Pb) in Fig. 5b. Analyses in TNL-1-1 were made in zircon grains with both concordant and heavily discordant ages ranging from 2992 to 1439 Ma. The data form a horizontal array with a very limited range of δ^18^O of 8.98 to 9.42‰. NDV-2 exhibits consistently higher values, with a range from 10.51 to 11.65‰. The oldest analysis from NDV-2 forms an outlier with a lower δ^18^O of 9.25. Data from both samples are much higher than mantle values (5.3 ± 0.3‰)^37^; for magmatic zircon these data at face value imply a contribution to the crystallising melt of low-temperature altered (i.e. supracrustal) material^7,38^.

### Lu-Hf

Hf isotope data are plotted as apparent zircon age (^207^Pb/^206^Pb) against the age-corrected ^176^Hf/^177^Hf ratio in Fig. 5c. TNL-1-1 shows a horizontal array extending from ca. 3060 to 1440 Ma at ca. 0.2807 that can be interpreted as a lead-loss trend (Lu/Hf = 0). A second cluster has apparent ages around ca. 2600 to 2350 Ga, also with discordant analyses falling on a lead-loss trend from this population. Two analyses from TNL-1-1 have higher initial ^176^Hf/^177^Hf ratios (0.28122 and 0.28175), with one ca. 1650 Ma analysis being a distinct outlier. NDV-2 has a single horizontal array ranging from ca. 3200 to 2700 Ma, with a cluster around 3.1 Ga that has an average composition just lower than that of CHUR. The array is compatible with a lead-loss trend. A single outlier falls above CHUR at 2.7 Ga, and at face value would represent a more juvenile composition outside of that expected from reworking of the 3100 Ma population alone.

### Trace elements

Titanium contents in zircon can be used as an indicator of the temperature of the melt during zircon crystallization^11,39,40^. This is most accurate in magmatic and metamorphic rocks where the activity of Ti can be assessed through the presence of Ti-phases such as rutile. In detrital rocks, as in this study here, the temperatures can only arbitrarily be used, as the Si and Ti activities during crystallisation that are required for the temperature calculation are not known. Using typical Si and Ti activities of 1 and 0.7, respectively, the Ti-in-zircon temperatures range from 767 to 853 °C for TNL-1-1 and from 708 to 959 °C for NDV-2. These temperatures are higher than the average compositions of both mafic and felsic rocks^12^. However, it should be noted that the measurement uncertainty on the Ti contents leads to an average temperature uncertainty of 30 °C, and that drastically changing the Si and Ti contents can lead to an up to 70 °C lowering in the temperature.

**Figure 6:**
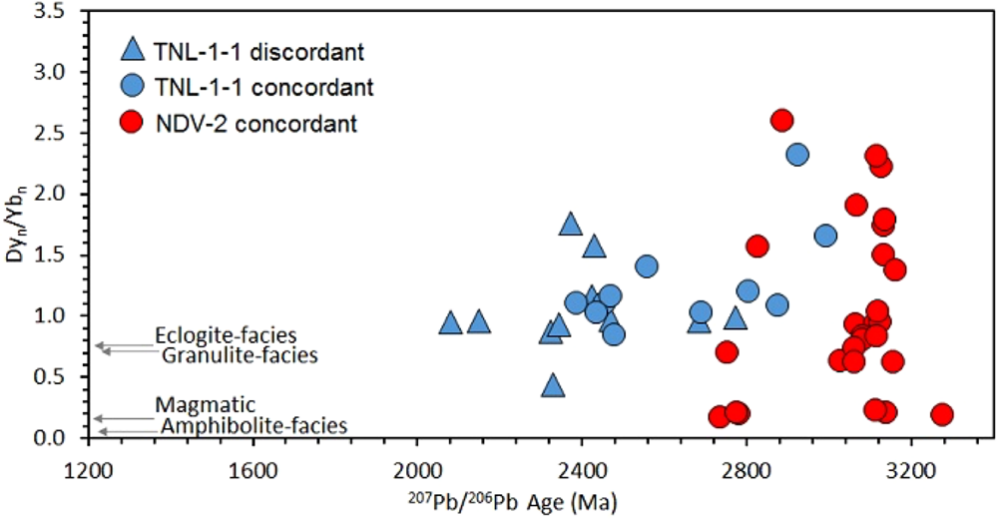
Chondrite normalised^64^ Dy/Yb ratios plotted against ^207^Pb/^206^Pb age (Ma). The mean Dy/Yb ratio for granulite-, eclogite- and amphibolite-facies zircon, along with magmatic zircon, is taken from^42^.

**Figure 7:**
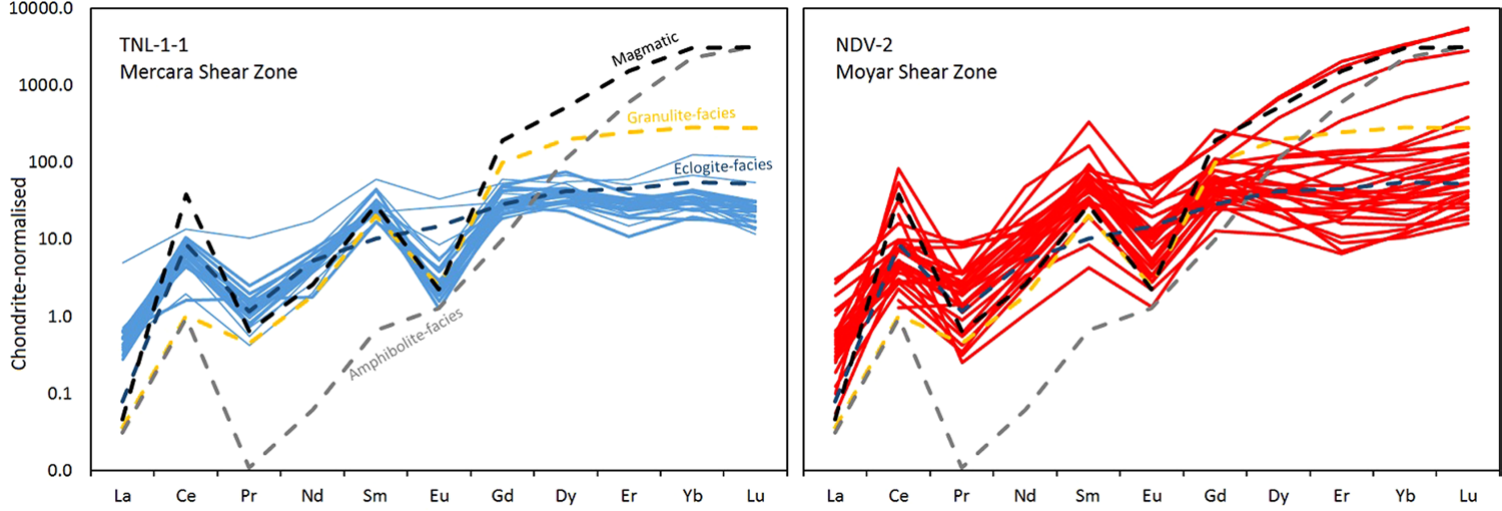
Chondrite-normalised^64^ REE profiles for TNL-1-1 and NDV-2, shown with profiles for granulite-, eclogite- and amphibolite-facies metamorphic zircon, along with magmatic zircon taken from^42^. Thin lines represent analyses with correlative discordant (>90%) U-Pb ages, and thicker lines represent those relating to concordant U-Pb data.

Chondrite-normalised Dy/Yb (i.e. MREE/HREE) and REE patterns are shown in Figs 6 and 7, respectively. The data for TNL-1-1 are tightly spread, and show distinct positive Ce and negative Eu anomalies that are typical for zircon formed in both magmatic settings and amphibolite- to granulite-facies metamorphic conditions. The MREE to HREE are relatively flat for all analyses, which is typical of granulite-facies zircon grown in the presence of HREE-sequestering minerals, primarily garnet^41,42^. Only one sample is an outlier, and lacks an Eu anomaly and also displays less depleted HREE. Sample NDV-2 has greater scatter in the data, but overall with similar patterns. A few analyses lack the HREE depletion, and have steeper MREE-HREE patterns typical of magmatic zircon. The MREE to HREE ratio, i.e. a monitor of HREE depletion typical of high-grade metamorphic zircon^42^, and plotted as chondrite normalized Dy/Yb, is shown against apparent ^207^Pb/^206^Pb age in Fig. 6. There is no correlation between HREE depletion and age, irrespective of the discordance of the age.

**Figure 8:**
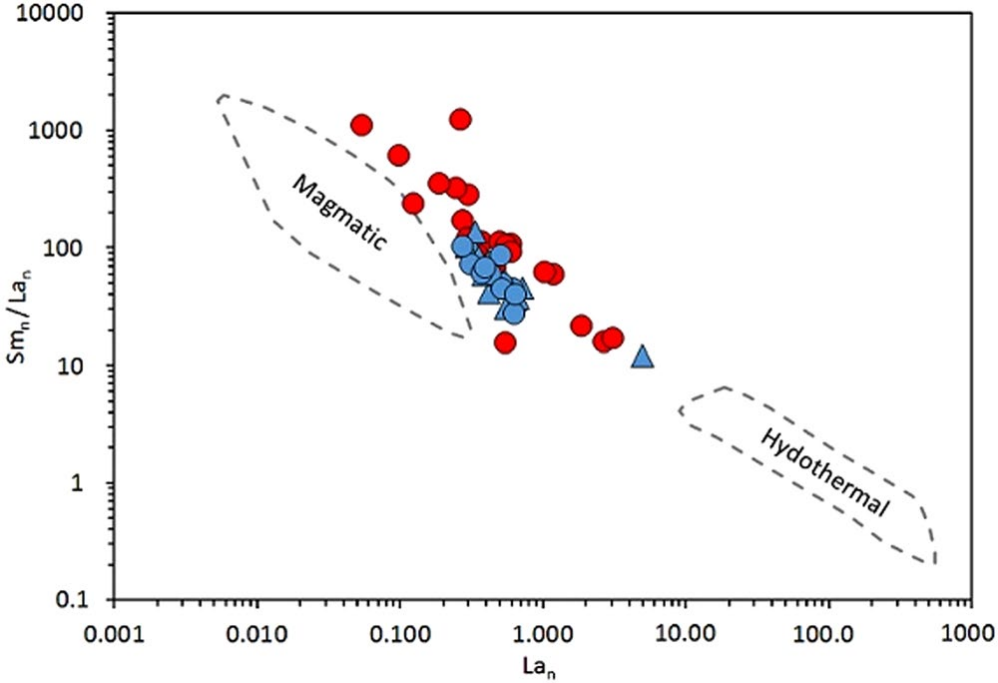
Chondrite-normalised^64^ La versus Sm/La. Fields for magmatic and hydrothermal zircon from^43^ are shown for comparison. Symbols follow that of Figs 5 and 6.

Light rare earth element (LREE) contents can be used to discriminate hydrothermal zircon from magmatic zircon, as LREE are typically enriched during hydrothermal alteration^43^. A plot of chondrite-normalised La versus Sm/La (Fig. 8) shows that the bulk of the data do not cross into the hydrothermal field.

## Discussion

For several reasons outlined below, we interpret the combined isotopic and elemental zircon data presented to reflect alteration and disturbance of zircon, particularly of the oxygen isotope system, rather than primary signatures formed during crystallization. First, we list the various lines of evidence, and this is followed by an evaluation of the potential processes responsible and the constraints on the timing of these processes.

The following lines of evidence indicate alteration of the zircon after their deposition as sedimentary units:

1. Significant discordance in the U-Pb system for TNL-1-1 is indicative of lead-loss, the timing of this being loosely constrained to early Neoproterozoic.
2. A lead-loss trend in U-Pb-Hf space (Fig. 5b), combined with concordant U-Pb data, implies an old (Neoarchaean-Palaeoproterozoic) lead-loss event affecting NDV-2.
3. Oxygen isotope data are remarkably consistent across each sample, and much higher than that predicted for zircon grains of this age (see Fig. 5b). Detrital zircon grains are predicted to exhibit variable δ^18^O signatures reflecting the variability among their source rocks, but this is not the case for both samples.
4. REE profiles are remarkably consistent for TNL-1-1, especially considering the large age range of ages, and show flat MREE-HREE implying the signature was imparted during conditions with growth of other HREE-bearing phases, typical of granulite-facies. NDV-2 is more scattered, with a few magmatic signatures, but mostly showing the same flat MREE-HREE pattern. Detrital zircon grains are predicted to have variable patterns reflecting their source regions, and would likely comprise both magmatic and metamorphic signatures.

After establishing that the zircon grains exhibit some alteration to their isotopic signatures, it is pertinent to ask the question, would these zircon grains be classified as metamorphic based on their appearance or Th/U ratios? In CL, sample TNL-1-1 has consistent weak zoning with only faint oscillatory patterns. The analysed domains are not from clear overgrowths but typically from central domains. The optical images (see Supplementary Information), indicate that no oxygen isotope analyses were located near cracks which can act as potential alteration pathways, and only two samples are clearly metamict. Th/U ratios are mixed, with the minority having classical metamorphic values of <0.1. Sample NDV-2 has a wider variety of CL textures, with some analyses being located on strongly oscillatory zoned domains that would be classified as magmatic. Optical imagery shows that two analyses were located on cracked domains, and roughly half the zircon grains analysed are potentially metamict. Th/U ratios are again mixed, with less than half being from metamorphic values. Thus, we can conclude that although there is some evidence in CL for TNL-1-1, and optically for NDV-2, overall the zircon grains would not generally be classified as metamorphic and not suffering from strong metamictisation. The importance of this will be returned to in the concluding remarks.

Oxygen isotopes, due to their slow diffusion in dry conditions^2,3^ are thought to be robust within zircon, even after high-grade metamorphism, and this has been evidenced by several studies^8,19,44^. The exception to this is zircon that has been altered by hydrothermal fluids, in this case, newly grown zircon can deviate from the primary signature, and trend towards that of the metamorphic fluid^45^. For this reason, studies that wish to observe only primary signatures will tend to exclude data that result from cracked or pitted domains, from zircon grains that have discordant U-Pb age data or from zircon with metamorphic textures. There is debate over the use of wet or dry diffusion data for zircon^42,46^, because most natural data have pointed to slow diffusion more compatible with dry diffusion rates^5,8,10^, but experimental data have shown that diffusion under high water activity would be much faster^2,3^. However, a recent study^46^ from the Ukrainian Shield interpreted a set of high δ^18^O analyses as due to modification under high temperatures (~700 °C) in hydrothermal conditions, advocating the fast rates of diffusion under wet conditions. The non-metamict nature of zircon grains of that study, the high δ^18^O, and the Archaean ages that distinguish them from global datasets, is similar to the zircon data we present here. The authors^46^ recognised zircon with modified oxygen isotope compositions which were altered by a process independent of crystallinity, state of metamictisation and CL texture, and that had caused limited Hf isotope disturbance. Given the similar characteristics of the zircon systematics in the present study, we consider the δ^18^O data of this present study at face value to indicate a similar case of fluid-assisted resetting of oxygen isotope signatures.

The U-Pb, Hf-isotope and elemental signatures can be used to further constrain this postulated fluid-assisted alteration. Ti-in-zircon temperatures of both samples are typical of amphibolite- to granulite-facies conditions. Given the uncertainty over the accuracy of these data without evidence for equilibrium during Ti-incorporation, equilibrium with a Ti-phase, and the unknown pressure during metamorphism^47,48^, along with the unknown nature of the primary zircon signatures, we cannot be certain whether these reflect alteration of the Ti contents.

REE patterns for both samples are mostly typical of granulite-facies zircon, i.e. zircon that has low HREE due to its sequestration in HREE-bearing phases such as garnet. Previous estimates on the metamorphic history of these metasedimentary belts suggest conditions up to granulite-conditions^22,26,34,35^, and the peak temperature of NDV-2 was estimated at 720–760 °C^22^. Diffusion of REE is slower than that of oxygen, and thus we may expect primary signatures to be preserved through metamorphism if the zircon is not recrystallised or reset. However, because of the general homogeneity of the REE signatures exhibited here, we consider them to be potentially obtained during subsequent metamorphism. In this case, the HREE depletion in comparison to magmatic zircon could thus be related to two mechanisms: (1) fluid-assisted resetting of the zircon during metamorphism under granulite-facies conditions, with HREE sequestering into other phases such as garnet; or (2) fluid-assisted alteration disturbed the REE unequally so that HREE were lost preferentially to LREE and MREE from the zircon. The mineral assemblage of both samples (muscovite + quartz ± chlorite ± garnet ± sillimanite ± rutile), and the potential granulite-facies temperatures recorded by both samples, indicate that the first option is viable. However, because HREE diffuse faster than LREE^13^, the second mechanism cannot be excluded.

As with the Ukrainian case study^46^, we conclude that disturbance of the oxygen isotope signatures and potentially the REE contents is likely to have occurred via rapid oxygen diffusion in hydrous conditions during high-temperature metamorphism. The timing of this metamorphic event can partly be tied down by the U-Pb-Hf data. The oxygen isotope disturbance affects zircon of all ages; for TNL-1-1, this disturbance therefore must be younger than the maximum depositional age of ca. 2.5 Ga, and is likely to have occurred during the strong lead-loss inducing event in the Neoproterozoic. For NDV-2, the lead-loss array in time-Hf space suggests that the 2.9 Ga ages are not primary, but record lead-loss from 3.0–3.1 Ga ages. Such lead-loss parallel to concordia is typical of Achaean high-grade rocks^49^, and makes it hard to date events precisely. The alteration can only be constrained in this case to equal or younger than 2.9 Ga. Since the two samples come from different shear-zones, there is potential that the fluid-assisted alteration occurred at different times within each sample. Therefore the 2.36 to 2.22 Ga metamorphism recorded in the nearby region^34^ is potentially responsible for altering sample NDV-2. Both samples reveal some retrogression to hydrous greenschist facies mineral assemblages. This presents a later post-peak metamorphic event that could potentially be related to zircon alteration; however, given the expected behaviour of oxygen isotope diffusion^2,3,5^, we suggest that this is unlikely and that earlier high temperature alteration is more appropriate.

In summary, we postulate that zircon from two different localities has experienced fluid-assisted oxygen isotope diffusion during high temperature conditions in the shear-zones surrounding the Coorg Block. Studies that have shown rapid fluid-assisted oxygen isotope diffusion are scarce^46^, and therefore this work adds weight to the argument that wet diffusion rates may be more applicable to zircon in some settings. Given that high-grade metamorphism and deformation are both typically associated with fluid circulation, it is perhaps surprising that such observations from nature have not already been made. Deciphering the exact processes of alteration, i.e. by wet diffusion alone, by micro-fractures or micro-porosity, or by elemental substitution, will require much higher spatial resolution data obtainable by methods such as Atom Probe Tomography^50,51^.

It is important to recognise the potential for disturbed zircon isotopic systematics in detrital zircon, because many studies use archives of mineral isotopic and/or elemental data to study Earth evolution^16–20,52^. Such studies may involve, for example, filtering of oxygen data to obtain primary zircon signatures by various means: (1) removing discordant U-Pb analyses; (2) removing analyses with metamorphic (<0.1) Th/U ratios; (3) removing metamorphic-zoned domains by CL imaging; (4) removing physically cracked zircon domains; and (5) removing hydrothermally altered (high LREE) domains. However, in the case study presented here, such filtering will not have removed the entire set of high-δ^18^O zircon grains. Thus, this study presents a cautionary tale against the use of detrital δ^18^O zircon compilations, where a high level of scrutiny of individual datasets is typically not achieved, and where deciphering primary from secondary characteristics is hampered by the lack of host rock constraints.

## Methods

Zircon oxygen isotope studies were conducted using a Cameca IMS1280 ion microprobe housed at the Institute of Geology and Geophysics, Chinese Academy of Sciences, Beijing. Detailed working conditions and analytical procedures are the same as those described by^53^. The Cs^+^ primary ion beam was accelerated at 10 kV, with an intensity of ca. 2 nA and rastered over a 10 μm area. The spot is about 20 μm in diameter. The instrumental mass fractionation factor (IMF) was corrected using Penglai zircon standard with (δ^18^O)_VSMOW_ = 5.31 ± 0.10‰^54^. Measured ^18^O/^16^O was normalized by using Vienna Standard Mean Ocean Water (VSMOW) compositions, and then corrected for the instrumental mass fractionation factor. The internal precision of a single analysis was generally better than 0.2‰ for ^18^O/^16^O ratio. Values of δ^18^O were standardized to VSMOW, and the δ^18^O values are reported in standard per mil notation

U-Pb geochronology, Lu-Hf isotope analyses and trace element analyses were conducted at the NERC Isotope Geoscience Laboratory (NIGL) in Nottingham, UK. U-Pb geochronology was measured using a Nu Attom single collector inductively coupled plasma mass spectrometry coupled to a New Wave Research (ESI) 193UC excimer laser ablation system, following procedures documented in^55^. Lu-Hf isotope analyses were conducted using the same laser coupled to a Thermo Scientific Neptune Plus multi-collector ICP-MS, following procedures outlined in^56^. Trace elements, including Ti contents, were conducted using the same set-up as for U-Pb. Procedures are modified from those previously described^57,58^. Data were collected in the following order: δ^18^O (ion microprobe), U-Pb (LA-SC-ICP-MS), Lu-Hf (LA-MC-ICP-MS), and trace elements (LA-SC-ICP-MS). All analyses were placed on top of each other, except for trace element pits, which were placed in the same zircon domain, as imaged by CL, if space allowed.

All data generated or analysed during this study are included in this published article (and its Supplementary Information files).

## Electronic supplementary material

Supplementary Information

Dataset 1

## References

[1] Cherniak DJ, Hanchar JH, Watson EB (1997). Diffusion of tetravalent cations in zircon. Contributions to Mineralogy and Petrology.

[2] Watson EB, Cherniak DJ (1997). Oxygen diffusion in zircon. Earth and Planetary Science Letters.

[3] Cherniak DJ, Watson EB (2003). Diffusion in zircon. Reviews in mineralogy and geochemistry.

[4] Hoskin PWO, Black LP (2000). Metamorphic zircon formation by solid‐state recrystallization of protolith igneous zircon. Journal of metamorphic Geology.

[5] Peck WH, Valley JW, Graham CM (2003). Slow oxygen diffusion rates in igneous zircons from metamorphic rocks. American Mineralogist.

[6] Kinny PD, Maas R (2003). Lu–Hf and Sm–Nd isotope systems in zircon. Reviews in Mineralogy and Geochemistry.

[7] Valley JW (2003). Oxygen isotopes in zircon. Reviews in mineralogy and geochemistry.

[8] Page F (2007). High-precision oxygen isotope analysis of picogram samples reveals 2 μm gradients and slow diffusion in zircon. American Mineralogist.

[9] Gerdes A, Zeh A (2009). Zircon formation versus zircon alteration—new insights from combined U–Pb and Lu–Hf *in-situ* LA-ICP-MS analyses, and consequences for the interpretation of Archean zircon from the Central Zone of the Limpopo Belt. Chemical Geology.

[10] Bowman JR (2011). Zircon U-Pb isotope, δ18O and trace element response to 80 my of high temperature metamorphism in the lower crust: Sluggish diffusion and new records of Archean craton formation. American Journal of Science.

[11] Watson EB, Harrison TM (2005). Zircon thermometer reveals minimum melting conditions on earliest Earth. Science.

[12] Fu BF (2008). Ti-in-zircon thermometry: applications and limitations. Contributions to Mineralogy and Petrology.

[13] Cherniak DJ, Hanchar JM, Watson EB (1997). Rare-earth diffusion in zircon. Chemical Geology.

[14] Cherniak DJ, Watson EB, Wark DA (2007). Ti diffusion in quartz. Chemical Geology.

[15] Kemp AIS, Hawkesworth CJ, Paterson BA, Kinny PD (2006). Episodic growth of the Gondwana supercontinent from hafnium and oxygen isotopes in zircon. Nature.

[16] Belousova EA (2010). The growth of the continental crust: constraints from zircon Hf-isotope data. Lithos.

[17] Dhuime B, Hawkesworth CJ, Cawood PA, Storey CD (2012). A change in the geodynamics of continental growth 3 billion years ago. Science.

[18] Iizuka T (2013). Evolution of the African continental crust as recorded by U–Pb, Lu–Hf and O isotopes in detrital zircons from modern rivers. Geochimica et Cosmochimica Acta.

[19] Spencer CJ (2014). Proterozoic onset of crustal reworking and collisional tectonics: Reappraisal of the zircon oxygen isotope record. Geology.

[20] Roberts NMW, Spencer CJ (2015). The zircon archive of continent formation through time. Geological Society, London, Special Publications.

[21] Santosh M (2015). An exotic Mesoarchean microcontinent: the Coorg Block, southern India. Gondwana Research.

[22] Santosh M (2016). Oldest rocks from Peninsular India: evidence for Hadean to Neoarchean crustal evolution. Gondwana Research.

[23] Santosh M, Maruyama S, Sato K (2009). Anatomy of a Cambrian suture in Gondwana: Pacific-type orogeny in southern India?. Gondwana research.

[24] Plavsa D (2012). Delineating crustal domains in Peninsular India: age and chemistry of orthopyroxene-bearing felsic gneisses in the Madurai Block. Precambrian Research.

[25] Collins AS, Clark C, Plavsa D (2014). Peninsular India in Gondwana: the tectonothermal evolution of the Southern Granulite Terrain and its Gondwanan counterparts. Gondwana Research.

[26] Amaldev T (2016). Mesoarchean convergent margin processes and crustal evolution: Petrologic, geochemical and zircon U–Pb and Lu–Hf data from the Mercara Suture Zone, southern India. Gondwana Research.

[27] Peucat JJ, Mahabaleswar B, Jayananda M (1993). Age of younger tonalitic magmatism and granulitic metamorphism in the South Indian transition zone (Krishnagiri area); comparison with older Peninsular gneisses from the Gorur–Hassan area. Journal of Metamorphic Geology.

[28] Peucat JJ (2013). The lower crust of the Dharwar Craton, Southern India: Patchwork of Archean granulitic domains. Precambrian Research.

[29] Sato K, Santosh M, Tsunogae T, Chetty TRK, Hirata T (2011). Subduction–accretion–collision history along the Gondwana suture in southern India: a laser ablation ICP-MS study of zircon chronology. Journal of Asian Earth Sciences.

[30] Samuel VO, Santosh M, Liu S, Wang W, Sajeev K (2014). Neoarchean continental growth through arc magmatism in the Nilgiri Block, southern India. Precambrian Research.

[31] Chetty TRK, Yellappa T, Santosh M (2016). Crustal architecture and tectonic evolution of the Cauvery Suture Zone, southern India. Journal of Asian Earth Sciences.

[32] Ishwar-Kumar C (2016). Mesoproterozoic suturing of Archean crustal blocks in western peninsular India: Implications for India–Madagascar correlations. Lithos.

[33] Sunil PS (2010). Crustal structure of the western part of the Southern Granulite Terrain of Indian Peninsular Shield derived from gravity data. Journal of Asian Earth Sciences.

[34] Yano M (2016). Ultrahigh-temperature metagabbros from Wynad: Implications for Paleoproterozoic hot orogen in the Moyar Suture Zone, southern India. Journal of Asian Earth Sciences.

[35] Yang Q-Y (2016). Melt-fluid infiltration in Archean suprasubduction zone mantle wedge: Evidence from geochemistry, zircon U–Pb geochronology and Lu–Hf isotopes from Wynad, southern India. Precambrian Research.

[36] Bhadra S, Nasipuri P (2017). Tectonothermal evolution of a garnet-bearing quartzofeldspathic gneiss from the Moyar shear zone, south India and its bearing on the Neoarchean accretionary tectonics. Lithos.

[37] Valley JW, Kinny PD, Schulze DJ, Spicuzza MJ (1998). Zircon megacrysts from kimberlite: oxygen isotope variability among mantle melts. Contributions to Mineralogy and Petrology.

[38] Valley JW (2005). 4.4 billion years of crustal maturation: oxygen isotope ratios of magmatic zircon. Contributions to Mineralogy and Petrology.

[39] Ferry JM, Watson EB (2007). New thermodynamic models and revised calibrations for the Ti-in-zircon and Zr-in-rutile thermometers. Contributions to Mineralogy and Petrology.

[40] Watson EB, Wark DA, Thomas JB (2006). Crystallization thermometers for zircon and rutile. Contributions to Mineralogy and Petrology.

[41] Rubatto D (2002). Zircon trace element geochemistry: partitioning with garnet and the link between U–Pb ages and metamorphism. Chemical geology.

[42] Rubatto D (2017). Zircon: the metamorphic mineral. Reviews in mineralogy and geochemistry.

[43] Hoskin PWO (2005). Trace-element composition of hydrothermal zircon and the alteration of Hadean zircon from the Jack Hills, Australia. Geochimica et Cosmochimica Acta.

[44] Chen Y-X (2011). Metamorphic growth and recrystallization of zircons in extremely 18 O-depleted rocks during eclogite-facies metamorphism: evidence from U–Pb ages, trace elements, and O–Hf isotopes. Geochimica et Cosmochimica Acta.

[45] Martin LAJ, Duchêne S, Deloule E, Vanderhaeghe O (2008). Mobility of trace elements and oxygen in zircon during metamorphism: consequences for geochemical tracing. Earth and Planetary Science Letters.

[46] Claesson S, Bibikova EV, Shumlyanskyy L, Whitehouse MJ, Billström K (2016). Can oxygen isotopes in magmatic zircon be modified by metamorphism? A case study from the Eoarchean Dniester-Bug Series, Ukrainian Shield. Precambrian Research.

[47] Ferriss EDA, Essene EJ, Becker U (2008). Computational study of the effect of pressure on the Ti-in-zircon geothermometer. European Journal of Mineralogy.

[48] Hofmann AE, Baker MB, Eiler JM (2014). Sub-micron-scale trace-element distributions in natural zircons of known provenance: implications for Ti-in-zircon thermometry. Contributions to Mineralogy and Petrology.

[49] Whitehouse MJ, Kemp AIS (2010). On the difficulty of assigning crustal residence, magmatic protolith and metamorphic ages to Lewisian granulites: constraints from combined *in situ* U–Pb and Lu–Hf isotopes. Geological Society, London, Special Publications.

[50] Peterman EM (2016). Nanogeochronology of discordant zircon measured by atom probe microscopy of Pb-enriched dislocation loops. Science Advances.

[51] Piazolo S (2016). Deformation-induced trace element redistribution in zircon revealed using atom probe tomography. Nature communications.

[52] Spencer CJ, Roberts NMW, Santosh M (2017). Growth, destruction, and preservation of Earth’s continental crust. Earth-Science Reviews.

[53] Li X-H (2010). Petrogenesis and tectonic significance of the ∼850 Ma Gangbian alkaline complex in South China: evidence from *in situ* zircon U–Pb dating, Hf–O isotopes and whole-rock geochemistry. Lithos.

[54] Li X‐H (2010). Penglai zircon megacrysts: a potential new working reference material for microbeam determination of Hf–O isotopes and U–Pb age. Geostandards and Geoanalytical Research.

[55] Roberts NMW, Thomas RJ, Jacobs J (2016). Geochronological constraints on the metamorphic sole of the Semail ophiolite in the United Arab Emirates. Geoscience Frontiers.

[56] Hopkinson TN (2017). The identification and significance of pure sediment-derived granites. Earth and Planetary Science Letters.

[57] Tapster S (2016). Rapid thermal rejuvenation of high-crystallinity magma linked to porphyry copper deposit formation; evidence from the Koloula Porphyry Prospect, Solomon Islands. Earth and Planetary Science Letters.

[58] Joosu L (2015). The REE-composition and petrography of apatite in 2Ga Zaonega Formation, Russia: The environmental setting for phosphogenesis. Chemical Geology.

[59] Chetty TRK, Mohanty DP, Yellappa T (2012). Mapping of shear zones in the Western Ghats, Southwestern part of Dharwar Craton. Journal of the Geological Society of India.

[60] Rubatto, D. & Gebauer, D. Use of cathodoluminescence for U-Pb zircon dating by ion microprobe: some examples from the Western Alps. In *Cathodoluminescence in geosciences* 373–400 (Springer Berlin Heidelberg, 2000).

[61] Griffin WL (2000). The Hf isotope composition of cratonic mantle: LAM-MC-ICPMS analysis of zircon megacrysts in kimberlites. Geochimica et Cosmochimica Acta.

[62] Bouvier A, Vervoort JD, Patchett PJ (2008). The Lu–Hf and Sm–Nd isotopic composition of CHUR: constraints from unequilibrated chondrites and implications for the bulk composition of terrestrial planets. Earth and Planetary Science Letters.

[63] Payne JL (2016). Strengths and limitations of zircon Lu-Hf and O isotopes in modelling crustal growth. Lithos.

[64] Sun S-S, McDonough WF-S (1989). Chemical and isotopic systematics of oceanic basalts: implications for mantle composition and processes” Geological Society, London, Special Publications.

