# Supplementary material for: Rapid oxygen diffusion during high temperature alteration of zircon
Source: Sci Rep. 2018 Feb 26;8:3661. doi: 10.1038/s41598-018-22016-2 (PMC5827751; doi:10.1038/s41598-018-22016-2)
Supplement: Supplementary file 1 — Supplementary Information [file 41598_2018_22016_MOESM1_ESM.docx]

# Supplementary Information for:

# Rapid oxygen diffusion during high temperature alteration of zircon

## Nick M W Roberts^1^, Qiong-Yan Yang^2^, M Santosh^2,3,4^

^1^NERC Isotope Geosciences Laboratory, British Geological Survey, Nottingham, NG12 5GG, UK

^2^School of Earth Sciences and Resources, China University of Geosciences Beijing, 29 Xueyuan Road, Beijing 100083, China

^3^Centre for Tectonics, Exploration and Research, University of Adelaide, Adelaide, SA 5005, Australia

^4^Department of Geology, Northwest University, Northern Taibai Str. 229, Xi'an 710069, China

## Figure S1:

Optical transmitted and reflected light photographs of zircons analysed for δ^18^O; pits after oxygen isotope ion microprobe analyses are visible.


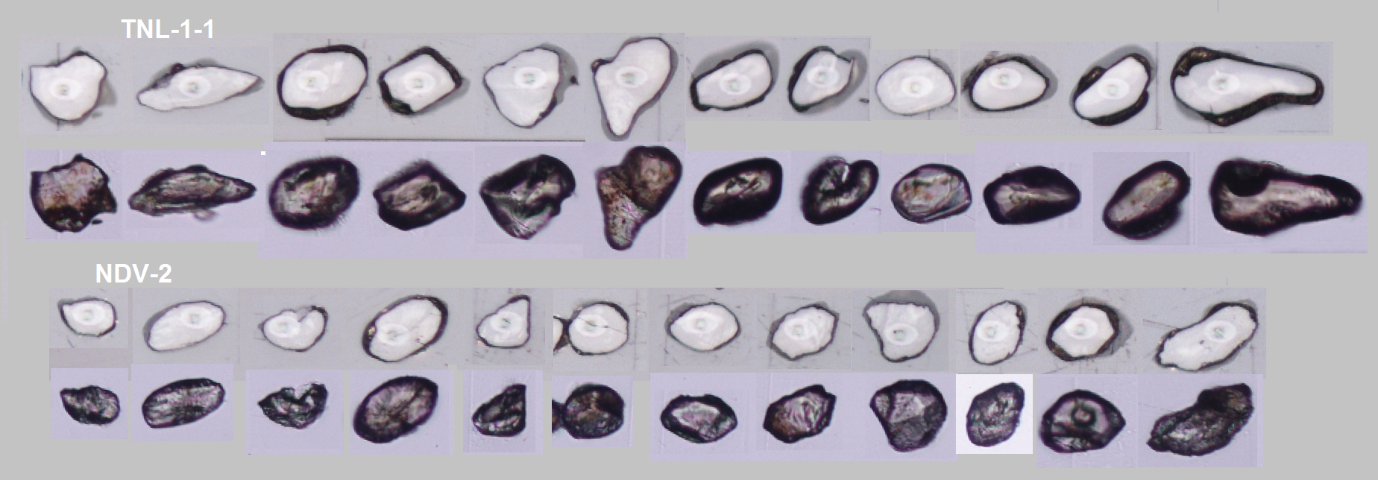


## Table S1:

Analytical conditions for LA-ICP-MS U-Pb analyses.

| **Laboratory & Sample Preparation** |  |
| --- | --- |
| Laboratory name | NERC Isotope Geosciences Laboratory Geochronology and Tracers Facility (NIGL-GTF) |
| Sample type/mineral | Zircon |
| Sample preparation | Conventional mineral separation, 1 inch resin mount, 1μm polish to finish |
| Imaging | CL |
| **Laser ablation system** |  |
| Make, Model & type | ESI/New Wave Research, UC193 |
| Ablation cell & volume | NWR TV2 |
| Laser wavelength (nm) | 193 nm |
| Pulse width (ns) | 4 ns |
| Fluence (J.cm^-2^) | 2.5 J.cm^-2^ |
| Repetition rate (Hz) | 5 Hz |
| Ablation duration (secs) | 20 secs |
| Ablation pit depth / ablation rate | 10μm pit depth, equivalent to 0.08μm/pulse |
| Spot diameter (μm) nominal/actual | 20 µm |
| Sampling mode / pattern | Static spot ablation |
| Carrier gas | 100% He in the cell, Ar make-up gas combined using a Y-piece 50% along the sample transport line to the torch. |
| Cell carrier gas flow (l/min) | 0.6 |
| **ICP-MS Instrument** |  |
| Make, Model & type | Nu Instruments, Attom, SC-ICP-MS |
| Sample introduction | Ablation aerosol |
| RF power (W) | 1300W |
| Make-up gas flow (l/min) | Sourced from Nu Instruments DSN-100 desolvating nebulizer. Neb pressure 24psi (estimated at 0.7l/min) Ar. |
| Detection system | Single MasCom multiplier |
| Masses measured | 202-208, 232, 235 |
| Integration time per peak/dwell times (ms); quadrupole settling time between mass jumps | 200 µs for ^202^Hg, ^204^X, ^206^Pb, ^208^Pb, ^232^Th  800 µs for ^207^Pb  1200 µs for ^235^U |
| Total integration time per output datapoint (secs) | ~0.3 ms |
| ‘Sensitivity’ as useful yield (%, element) | 0.015% U  ((#ions detected/#atoms sampled)*100; Schaltegger *et al.* 2015) |
| IC Dead time (ns) | 15 ns |
| **Data Processing** |  |
| Gas blank | 60 second on-peak zero subtracted |
| Calibration strategy | 91500 used as primary reference material, Plesovice & GJ1 used as secondaries/validation |
| Reference Material info | 91500 (Wiedenbeck et al., 1995)  Plešovice (Sláma et al., 2008)  GJ1 (Horstwood et al., 2016) |
| Data processing package used / Correction for LIEF | Nu Instruments TRA software and in-house spreadsheet for data normalization, uncertainty propagation and age calculation. LIEF correction assumes reference material and samples behave identically. |
| Mass discrimination | Standard sample bracketing using reference material |
| Common-Pb correction, composition and uncertainty | No common-Pb correction applied to the data. |
| Uncertainty level & propagation | Ages are quoted at 2*s* absolute, propagation is by quadratic addition. Reproducibility and age uncertainty of reference material and common-Pb composition uncertainty are propagated where appropriate according to Horstwood et al. (2016). |
| Quality control / Validation | **Secondary material results:**  **91500**  207Pb/206Pb = 1063 ± 8 Ma (2s) n=34/36  206Pb/238U = 1069 ± 5 Ma (2s) n=36/36  **GJ-1**  207Pb/206Pb = 613 ± 5 Ma (2s) n=33/36  206Pb/238U = 602 ± 2 Ma (2s) n=36/36 |
| **Other information** |  |

## Table S2:

Analytical conditions for LA-ICP-MS Hf isotope analyses.

| **Laboratory & Sample Preparation** |  |
| --- | --- |
| Laboratory name | NERC Isotope Geosciences Laboratory Geochronology and Tracers Facility (NIGL-GTF) |
| Sample type/mineral | Zircon |
| Sample preparation | Zircon in 1-inch epoxy mount. |
| Imaging | CL |
| **Laser ablation system** |  |
| Make, Model & type | ESI NWR 193UC |
| Ablation cell & volume | NWR TV2 |
| Laser wavelength (nm) | 193nm |
| Fluence (J.cm^-2^) | 7 |
| Repetition rate (Hz) | 10 |
| Ablation duration (secs) | 30 |
| Spot diameter (μm) nominal/actual | 30 |
| Sampling mode / pattern | Static |
| Carrier gas | He |
| Cell carrier gas flow (l/min) | 0.7 |
| **ICP-MS Instrument** |  |
| Make, Model & type | Thermo Scientific Neptune Plus, MC-ICP-MS |
| Sample introduction | Aridus II (U-series one) |
| RF power (W) | 1400W |
| Make-up gas flow (l/min) | #N/A |
| Detection system | Faraday |
| Masses measured | 172-180 |
| Integration time per peak/dwell times (ms); quadrupole settling time between mass jumps | 1 sec |
| Total integration time per output datapoint (secs) | 1 sec |
| ‘Sensitivity’ as useful yield (%, element) | 1% Hf using x-cone and H sampler |
| IC Dead time (ns) | #N/A |
| **Data Processing** |  |
| Gas blank | on-peak zero subtracted |
| Calibration strategy | Normalised to JMC475 and internally Hf corrected |
| Data processing package used / Correction for LIEF | Iolite |
| Mass discrimination | Internal Hf correction with measured or pre-calibrated Yb correction |
| Common-Pb correction, composition and uncertainty | #N/A |
| Uncertainty level & propagation | Iolite processing uses excess variance calculation |
| Quality control / Validation | **JMC475**  ^176^Hf/^177^Hf = 0.282162 ± 0.000003 (95% conf.)  **91500**  ^176^Lu/^177^Hf = 0.00031109 ± 0.00000047 (95% conf.)  ^176^Hf/^177^Hf = 0.282306 ± 0.000012 (95% conf.)    **GJ-1**  ^176^Lu/^177^Hf = 0.0002276 ± 0.0000033 (95% conf.)  ^176^Hf/^177^Hf = 0.282009 ± 0.000013 (95% conf.) |
| **Other information** |  |

## Table S3:

Analytical conditions for trace element zircon analyses

| **Laboratory & Sample Preparation** |  |
| --- | --- |
| Laboratory name | NERC Isotope Geosciences Laboratory Geochronology and Tracers Facility (NIGL-GTF) |
| Sample type/mineral | Zircon |
| Sample preparation | Conventional mineral separation, 1 inch resin mount, 1μm polish to finish |
| Imaging | CL |
| **Laser ablation system** |  |
| Make, Model & type | ESI/New Wave Research, UC193 |
| Ablation cell & volume | NWR TV2 |
| Laser wavelength (nm) | 193 nm |
| Pulse width (ns) | 4 ns |
| Fluence (J.cm^-2^) | 2.5 J.cm^-2^ |
| Repetition rate (Hz) | 7 Hz |
| Ablation duration (secs) | 15 secs |
| Ablation pit depth / ablation rate | 10 μm pit depth, equivalent to 0.08μm/pulse |
| Spot diameter (μm) nominal/actual | 25 µm |
| Sampling mode / pattern | Static spot ablation |
| Carrier gas | 100% He in the cell, Ar make-up gas combined using a Y-piece 50% along the sample transport line to the torch. |
| Cell carrier gas flow (l/min) | 0.6 |
| **ICP-MS Instrument** |  |
| Make, Model & type | Nu Instruments, Attom, SC-ICP-MS |
| Sample introduction | Ablation aerosol |
| RF power (W) | 1300W |
| Make-up gas flow (l/min) | Sourced from Nu Instruments DSN-100 desolvating nebulizer. Neb pressure 24psi (estimated at 0.7l/min) Ar. |
| Detection system | Single MasCom multiplier. |
| Masses measured | ^31^P, ^44^Ca, ^49^Ti, ^93^Nb, ^139^La, ^140^Ce, ^141^Pr, ^146^Nd, ^149^Sm, ^153^Eu, ^157^Gd, ^163^Eu, ^166^Er, ^172^Yb, ^175^Lu, ^181^Ta, ^232^Th, ^238^U |
| Integration time per peak/dwell times (ms); quadrupole settling time between mass jumps | Linkscan Mode  200 ms per total sweep of mass range |
| Total integration time per output datapoint (secs) | 0.2s |
| ‘Sensitivity’ as useful yield (%, element) | 0.015% U  ((#ions detected/#atoms sampled)*100; Schaltegger *et al.* 2015) |
| IC Dead time (ns) | 15 ns |
| **Data Processing** |  |
| Gas blank | 60 second on-peak zero subtracted |
| Calibration strategy | 91500 used as primary reference material, ^29^Si for internal standard |
| Reference Material info | 91500 (Wiedenbeck et al., 1995) |
| Data processing package used / Correction for LIEF | Iolite |
| Mass discrimination | n/a |
| Common-Pb correction, composition and uncertainty | n/a |
| Uncertainty level & propagation | All uncertainties quoted at 2s. Reproducibility of reference material propagated where appropriate. |
| Quality control / Validation | GJ-1 within 20% of long-term laboratory values. |
| **Other information** |  |

Horstwood, M. S. A., et al. Community‐Derived Standards for LA‐ICP‐MS U‐(Th‐) Pb Geochronology–Uncertainty Propagation, Age Interpretation and Data Reporting. *Geostandards and Geoanalytical Research* **40**, 311-332 (2016).

Schaltegger, U., Schmitt, A. K. & Horstwood, M. S. A. U–Th–Pb zircon geochronology by ID-TIMS, SIMS, and laser ablation ICP-MS: recipes, interpretations, and opportunities. *Chemical Geology* **402** 89-110 (2015).

Sláma, J. et al. Plešovice zircon—a new natural reference material for U–Pb and Hf isotopic microanalysis. *Chemical Geology* **249**, 1-35 (2008).

Wiedenbeck, M. A. P. C., et al. Three natural zircon standards for U‐Th‐Pb, Lu‐Hf, trace element and REE analyses. *Geostandards and Geoanalytical Research* **19**, 1-23 (1995).
